# Supplementary figures and images for: Risk screening methods for extreme heat: Implications for equity-oriented adaptation
Source: PLoS One. 2020 Nov 4;15(11):e0240841. doi: 10.1371/journal.pone.0240841 (PMC7641348; doi:10.1371/journal.pone.0240841)

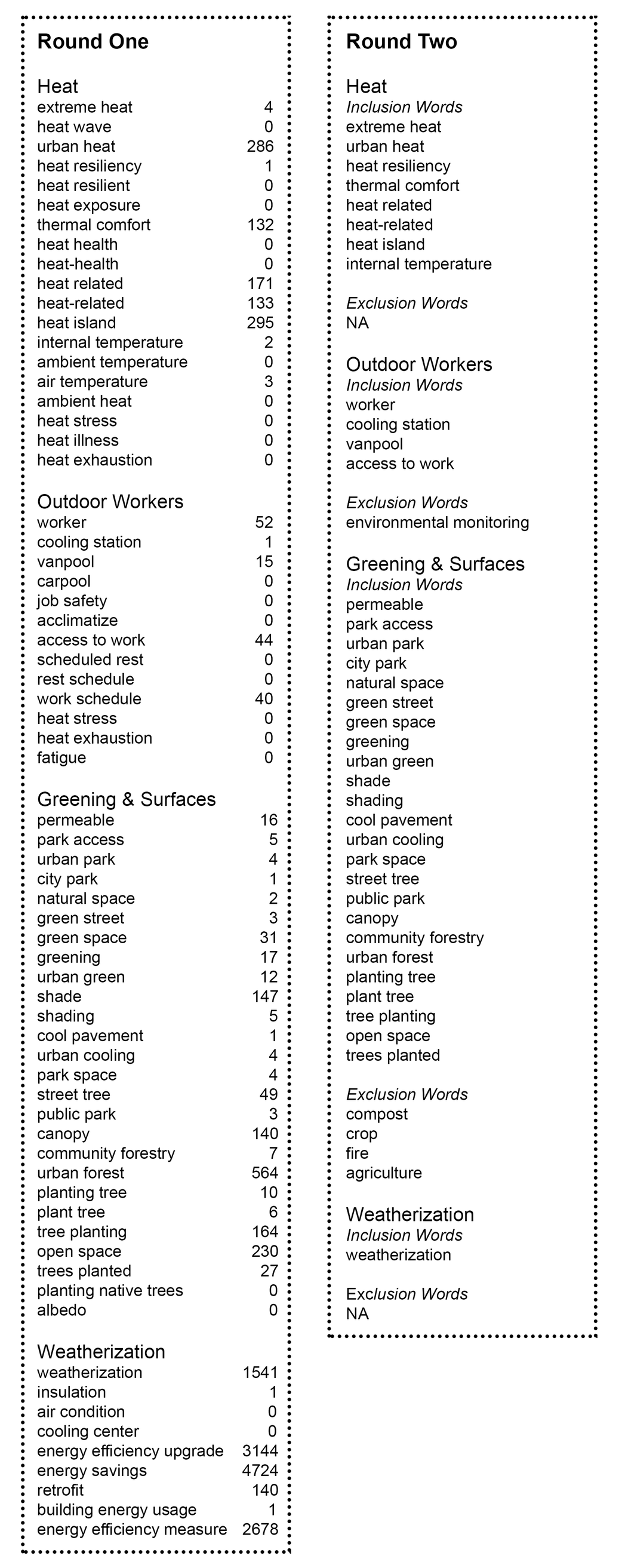

Supplement: S1 Fig — The table displays the first and second rounds of the keyword phrases used to screen for heat-related CCI tract interventions. Both rounds are split up into four adaptation relevant categories: heat specific, outdoor workers, greening and surfaces, and weatherization. The first round (left box) is the first iteration of phrases considered. The total number of interventions found by each phrase is listed to its right. The second round includes the inclusion and exclusion keyword phrases that were determined to produce heat relevant interventions (right box). (TIF) [file pone.0240841.s001.tif]

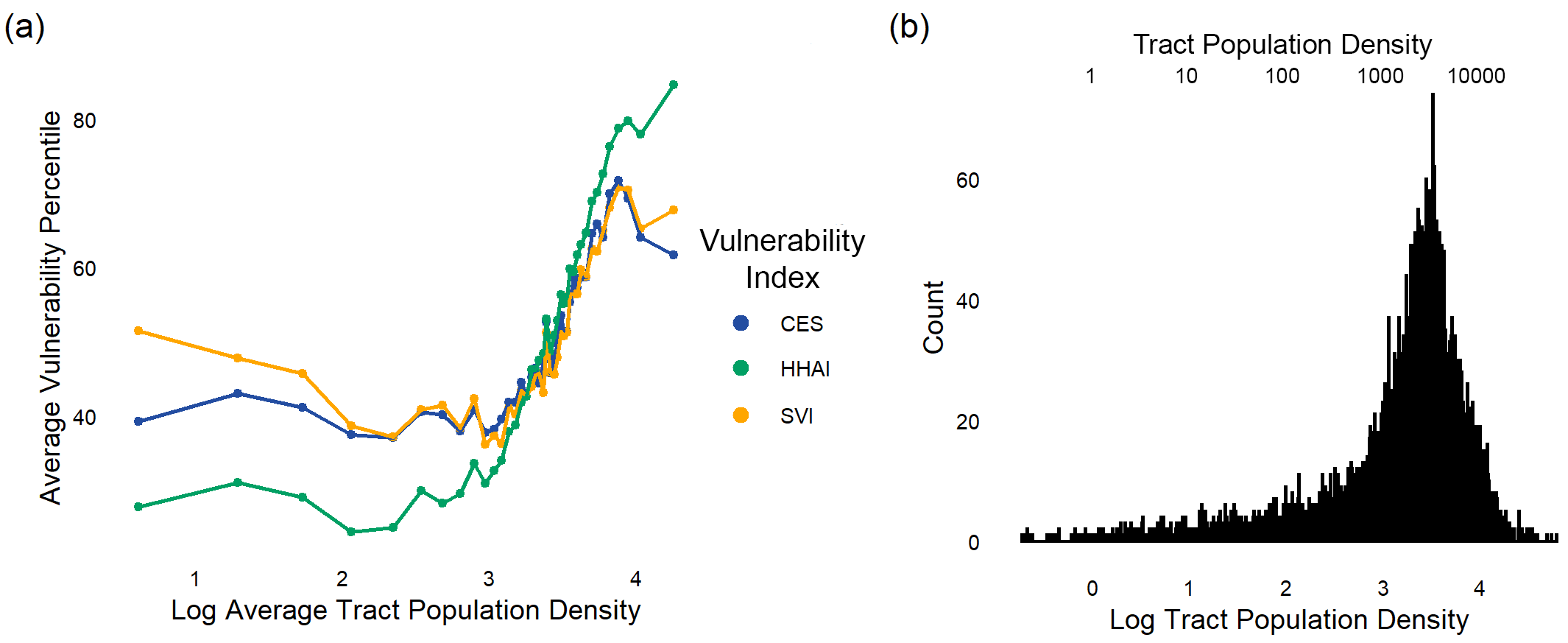

Supplement: S2 Fig — (a) Average vulnerability percentile (CES, HHAI, SVI) is plotted by log average population density of tracts (people per square kilometer, bin width of 2.5 percentiles, log scale). (b) This plot shows the frequency of different population densities across California’s census tracts (people per square kilometer, log scaled x-axis). (TIF) [file pone.0240841.s002.tif]

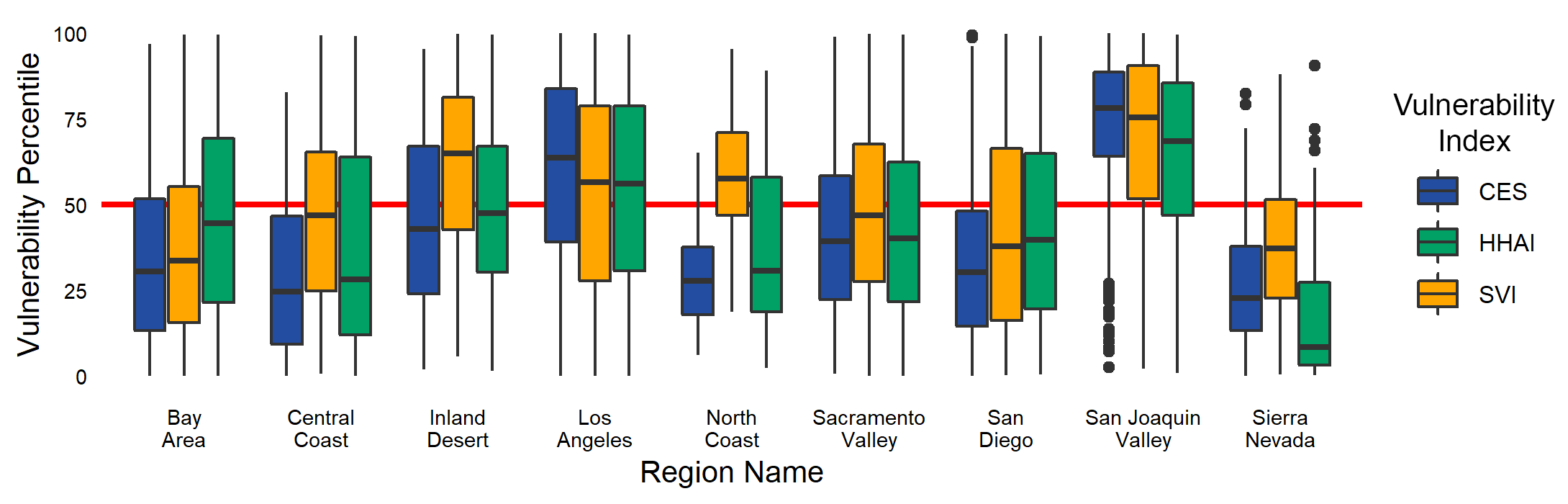

Supplement: S3 Fig — Each boxplot represents the distribution of census tract vulnerability percentiles for a region. Here we consider three indices: CES (blue), HHAI (green), and SVI (yellow). The red line represents the median. (TIF) [file pone.0240841.s003.tif]

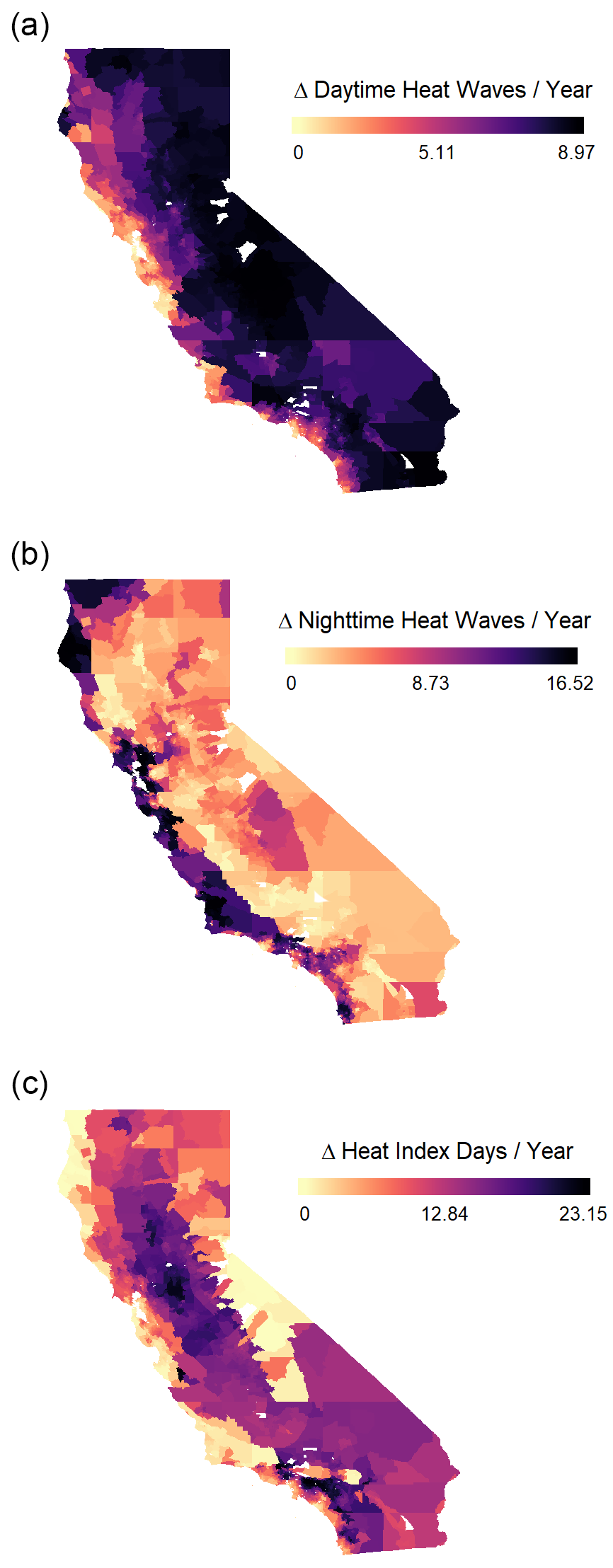

Supplement: S4 Fig — Plots display the increase in daytime heat waves (a), nighttime heat waves (b), and heat index days (c) for the state of California between the current (2006–2025) period and the future (2040–2059) period. For each metric of extreme heat, the projected change under RCP 4.5 is indicated based on the average of four climate models: HadGEM2-ES, CNRM-CM5, CanESM2, and MIROC5. (TIF) [file pone.0240841.s004.tif]
