# Supplementary material for: Risk screening methods for extreme heat: Implications for equity-oriented adaptation
Source: PLoS One. 2020 Nov 4;15(11):e0240841. doi: 10.1371/journal.pone.0240841 (PMC7641348; doi:10.1371/journal.pone.0240841)
Supplement: Attachment — Submitted filename: Response to Reviewers.docx [file pone.0240841.s005.docx]

**Point-by-point response to review of “Risk screening methods for extreme heat: implications for equity-oriented adaptation” (formerly titled “Equity-oriented adaptation to extreme heat in California”)**

**Structure of this file:** In this file, we have reproduced the tailored commentary from the editor’s letter (black italic font), including the editor’s summary, referee feedback, and production queries. We outline the changes made to the manuscript or provide additional background for each point raised (blue font). Line numbers referenced in this response correspond with the updated manuscript, not the original submission.

**Journal Requirements:**

*We note that Figures 1, 2 and supporting figure 1 in your submission contain map images which may be copyrighted… We require you to either (a) present written permission from the copyright holder to publish these figures specifically under the CC BY 4.0 license, or (b) remove the figures from your submission.”*

Author response: All data used to generate the maps originated from publicly available datasets and therefore do not require copyright permission. In Figures 1, 2 and Supporting Figure 4 spatial polygons come from the 2010 census tract boundaries dataset (<https://www.census.gov/geographies/mapping-files/time-series/geo/carto-boundary-file.2010.html>). Vulnerability data mapped in Figures 1 and 2 come from three publicly available datasets: CES is from the California Office of Environmental Health Hazard Assessment (<https://oehha.ca.gov/calenviroscreen/report/calenviroscreen-30>), SVI is from the Center for Disease Control and Prevention’s Agency for Toxic Substances and Disease Registry (<https://svi.cdc.gov/>), and HHAI is from California’s Fourth Climate Change Assessment and managed by the California Energy Commission (<https://www.cal-heat.org/>). Project data were downloaded from California Air Resources Board Website (<https://ww3.arb.ca.gov/cc/capandtrade/auctionproceeds/communityinvestments.htm>) and from the California Climate Investments website (<https://webmaps.arb.ca.gov/ccimap/>). Temperature and humidity data used in the supporting figure 1 maps originate from the Scripps Institute of Oceanography. Temperature data were downloaded through Cal-Adapt, a website managed by the California Energy Commission (<https://cal-adapt.org>) and humidity data were downloaded directly from the LOCA climate projections website (<http://loca.ucsd.edu>).

**Reviewer #1:**

*This paper uses three vulnerability indices for California to show how census-tract assessments of vulnerability are sensitive to the choice of vulnerability index. The paper further investigates how adaptation projects are distributed across census tracts, and shows how well the projects are meeting the target of helping vulnerable populations. The findings reveal that relying on a single index (CES) in the distribution of these projects could leave out some vulnerable communities that are shown to be vulnerable by other indices other than the CES. Overall, I found this paper to be extremely well written and informative. This is an important and timely topic and should be of interest to many. This work clearly has applications to climate change adaptation and planning in California. I see no major issues with the article, and I think it is acceptable for publication after addressing a few minor comments listed below.*

Author response: We appreciate the reviewer’s comments and interpretation of our analysis.

*Line 190: RCP 4.5 is a low-emissions scenario that assumes aggressive action to curb CO2 emissions. Under this scenario, radiative forcing from CO2 would stabilize before the end of the century, however, we are not on track to meet this goal. It would be useful to mention that the projections used in this study are likely to underestimate future heat impacts due to climate change, especially at the end of the century.*

Author response: Indeed, at present globally, we are not on track to meet the moderate emissions scenario (RCP 4.5) elected in this study, and under this scenario impacts are likely underestimated in the long term if emissions reductions do not occur. We note, additionally, that projections across scenarios do not vary substantially for the near-term timeframe focused upon in our analysis. However, it is important to prevent misinterpretation, and we now provide clarity by including the following text:

Lines 211-216: “**We used a moderate emissions scenario only (Representative Concentration Pathway 4.5) given overlapping climate projections across scenarios over the next few decades (43). By the end of the century, however, divergence across scenarios is more substantial. At present, global emissions and greenhouse gas mitigation efforts are not in line with the moderate trajectory, and without increasing stringency of emissions reductions, global warming and associated climate change impacts would exceed this trajectory in the longer term.**”

*Line 195: What is the source of the Tmin and Tmax temperature data?*

Author response: Tmin and Tmax data are from the Localized Constructed Analog downscaled developed by the Scripps Institute of Oceanography, specifically under the leadership of Dr. David Pierce. Data were downloaded via the Cal-Adapt API portal (<https://cal-adapt.org>). Below is an updated excerpt (lines 183-186) from the manuscript stating the source of the temperature data:

Lines 202-206: “We used Localized Constructed Analog downscaled Coupled Model Intercomparison Project 5 temperature data downloaded from Cal-Adapt, a government run API portal, and humidity data from the University of California San Diego’s archive (44–47). **We downloaded daily maximum temperature (Tmax), minimum temperature (Tmin), and minimum relative humidity data for each tract from these sources.”**

*Lines 460-463: You mention that the HHAI is the only index that is heat specific. This is an important point, but it is unclear from Table 1 how heat is included in this index.*

Author response: HHAI is a heat focused index because it is comprised of indicators that are considered relevant for determining heat-related impacts. We recognize that the classification of HHAI as heat specific is subtle and may not be clear to many readers. We include the following text to provide clarity:

Lines 338-340: “**HHAI includes three indicators that are important to characterizing urban-specific heat vulnerabilities but are less relevant for rural communities: impervious surface coverage, tree canopy coverage, and the change in urbanized land (Table 1).**”

**Reviewer #2:**

*Overview. This is a methodologically-oriented article that develops and applies a rigorous, if not entirely novel, comparative approach to three different heat vulnerability indices in California. It makes the important point that models matter and that designation of areas as vulnerable to extreme heat depends greatly on the type of index used. The article applies this analysis to a critique of state climate adaptation funding.*

Author response: We thank the reviewer for their overview of our analysis and perspective.

*The paper provides an excellent review of the literature on heat vulnerability but does not situate itself in the literature of the construction and comparison of indicators (with the exception of references 54 and 55—but these are referenced only in passing and only in the conclusion. This discussion should be brought up into the lit review and further developed. This detracts from the value of the paper in contributing to this growing field. Adding this component to the literature review could help the authors identify what is novel about their methods and how they could be fine-tuned to push the boundaries of the field .Two pieces that may be useful in this regard are:*

*• James Sadd et al. 2014. R9 RARE Final Project Report Title: Partnering with Environmental Agencies and Communities to Pilot Use of the Environmental Justice Screening Method (EJSM) Cumulative Impacts Tool.*

*US EPA Region 9.*

*• Liévanos, Raoul. 2018. Retooling CalEnviroScreen: Cumulative pollution burden and race-based environmental health vulnerabilities in California. International journal of environmental research and public health, 15(4), 762.*

Author response: We agree with the reviewer that the study would benefit from a bolstered literature review of the construction and comparison of indicators in the background section. We also thank the reviewer for the recommended articles, which are key sources. Below is an updated excerpt from the manuscript that addresses the comment:

Lines 126-151: “Vulnerability indices are often used to identify which populations are most susceptible to impacts from environmental hazards, but there are important differences and uncertainties across metrics (27). **Previous research demonstrated that data scaling, normalization, transformation, weighting, and aggregation techniques vary widely in index construction and that different design decisions and spatial resolutions impact measured outcomes (28–30). A commonly applied index-construction technique is principal component analysis and factor weighting. Heat-specific vulnerability indices similarly differ in indicator weightings and selection (26,31,32). The implications of different vulnerability index definitions and measurement methods as well as their consequences in guiding policy and funding are incompletely understood. Multiple studies agree on the importance of supplementing indices and high-level assessments of vulnerability and environmental justice with “ground-truthing,” which validates and embeds context into the process (33–35).**

California is a vast and diverse state, and it is unlikely that a single vulnerability definition could encompass all nuances relevant across communities and the hazards they face. California’s Fourth Climate Change Assessment Climate Justice Report touched on the limitations of CES in its analysis, comparing it to the Healthy Places Index (36). CEJA released a report focused on the use of CES in various environmental justice projects throughout the state, highlighting recommended uses of CES in policy making and implementation decisions (11). **Finally, an EPA-sponsored Regionally Applied Research Effort project used multiple environmental justice screening indices and ground-truthing methods to develop a regional understanding of cumulative pollution exposure in California’s San Joaquin Valley (33).** This study fortifies and expands on these projects. Furthermore, we aim to assist in the development of “more robust mapping tools,” an identified knowledge gap, by strengthening knowledge of potential impacts and biases that could result from using vulnerability indices in policy development and climate adaptation decision making (36).”

*One critique of CES (that can apply to the SVI and HHAI) is that as a state-wide tool, it is less sensitive to regional variations. That is, heat vulnerability can be the result of different factors in different environments and also require different adaptation strategies. The paper takes this up at line 322, but this section would be expanded upon. What is it about each tool that produces this pattern? Breaking out their analysis by region (exploring whether the tools different in their ability to identify high heat vulnerable communities) would provide some useful data for state agencies charged with using them.*

Author response: The reviewer highlights the important point that geographic variability shapes the determinants of vulnerability and that therefore some indices may be better equipped to characterize some communities’ risk than others. We add additional insight to highlight this fact and to better tackle why the indices are producing different patterns.

Lines 338-346: **“HHAI includes three indicators that are important to characterizing urban-specific heat vulnerabilities but are less relevant for rural communities: impervious surface coverage, tree canopy coverage, and the change in urbanized land (Table 1). SVI excludes environmental, pollution, and health related indicators, which may make it better able to characterize the drivers of vulnerability in low population density tracts (Table 1 and S2 Fig). Beyond urban versus rural heat differences, the indices do not consider regional environmental and climatic differences that influence how communities experience heat and therefore how they should adapt. For example, prior work has shown that coastal winds can play a significant role in heat exposure for the inland tracts of the South Coast (57).”**

Given that two of the indices, SVI and HHAI, are derived from principle component analysis, it is difficult to fully capture the influences of individual indicators. To better highlight the regional differences between indicators we include a new supplemental figure (S3 Fig), reproduced below. It helps elucidate dynamics occurring in many of the rural regions such as the Sierra Nevada, North Coast, Inland Desert, and Central Coast regions, which have the largest discrepancies between the median vulnerability percentile scores across the indices studied.

Lines 835-838: **S3 Fig: Indices vary in how they weigh vulnerability across California’s regions. Each boxplot represents the distribution of census tract vulnerability percentiles for a region. Here we consider three indices: CES (blue), HHAI (green), and SVI (yellow). The red line represents the median.**

We include the following text to support the new figure:

Lines 374-375: **“**No tracts in these regions are considered vulnerable by all three, **and of the tracts that are designated as vulnerable, SVI is the prevailing index for the designation (S3 Fig).”**

We also include the following text in the discussion:

Lines 531-533: “**This work demonstrates that some indices may characterize some regions better than others, and that there is likely no “correct” index that can balance the unique geospatial drivers of vulnerability.”**

*The article notes that CES is not a heat vulnerability/ climate change risk tool but could make a stronger point about the challenges for the state in using a general purpose tool for a specific purpose. This is increasingly challenging as CES has been tasked to cover more and more policy areas, from toxic waste management, to air quality management, to drinking water. The article could help argue that while holistic tools like CES are very valuable for directing resources to address overall vulnerability/ disadvantage, more context-specific tools (or perhaps customized modules) will be needed.*

Author response: One of the main critiques made in this paper, we agree, is the use of CalEnviroScreen for topics outside of its design scope. To emphasize this point further, following the reviewer’s comment, we added the following text:

Lines 519-535: “There are limits to the effectiveness of using a single index to guide a fund with projects and communities as diverse as those under the CCI program. **At present, CES is and has been used across state agencies for a variety of purposes beyond the GGRF, including but not limited to an abandoned underground storage tank initiative by the State Water Resources and Control Board, allocation of renewable energy by the California Public Utilities Commissions, and a project for active transportation by the California Department of Transportation (11).** Prior research has challenged the use of indices for broad and multifaceted applications (59) that fail to integrate contextual differences (60) or to have a clearly defined purpose (61). **Further, race-based indicators, which have been shown to be uniquely associated with environmental justice due to structural discriminatory legacies, were removed to make the tool legally viable for multiple agencies (62).** Although CES is responsive to environmental injustices, by itself it does not fully capture the climate justice adaptation problems that come with escalating extreme heat exposure. **This work demonstrates that some indices may characterize some regions better than others, and that there is likely no “correct” index that can balance the unique geospatial drivers of vulnerability. A general, statewide tool could be useful for high-level screening, but context-specific tools that can better capture and reflect the nuances of the specific problem in question and local variabilities are also needed.**”

*The discussion section acknowledges that no matter how sophisticated, tools like CES, SVI, and HHAI are, they are best used as screening methods (indeed, there is a tool by developed for the California Air Resources Board called the Environmental Justice Screening Method – see above report by Jim Sadd) that can flag areas that require more in-depth and ground-level assessment. While this is expensive to implement, it is important for public agencies not to rely solely on these “30,000 foot” models and instead commitment to on-site ground-truthing. This point could made more strongly and in the introduction instead of only at the end of the paper.*

Author response: The point that agencies should not purely rely on “30,000 foot” models that do not include ground-truthing is an important one. Further, given that previous studies share this finding, we agree that it should be elevated and further emphasized in the background section of the paper. The following was added to the background section to address the reviewer’s comment, creating cohesiveness with the discussion’s treatment of similar themes:

Lines 134-137: “**Multiple studies agree on the importance of supplementing indices and high-level assessments of vulnerability and environmental justice with “ground-truthing,” which validates and embeds context into the process (33–35).”**

*Finally, the paper does not directly address of equity-oriented adaptation” as promised in its title. That is, it does not study adaptation policies or practices per se, but only the tools that can help inform adaptation strategies. Because this would require a significant design of the paper, I would suggest changing the title as opposed to doing too much to align with it. However, the paper could discuss the ways in which these tools serve to direct funds to certain kinds of adaptation projects.*

Author response: Although the study does include an analysis of AB1550, we agree that a change in title could be made to better reflect the focus of the study. We have changed the title to the following:

**Risk screening methods for extreme heat: implications for equity-oriented adaptation**

*Lines 79-80. The paper makes the important point that many community advocates dislike the stigmatizing quality of terms such as disadvantaged or vulnerable. Could the authors suggest some alternatives?*

Author response: The use of language that supports environmental communities rather than minimizes them is pivotal and community-led research is needed to tackle this knowledge gap. We add the following to the future work section and provide this statement in the conclusion. This is work we as authors seek to explore in future projects:

Lines 596-597: **“Scholarship employing community-engaged methods (71) is needed to research framings and language that reflect how communities want to be represented in policy and research.”**

*Line 190: Define “Representative concentration pathway 4.5”*

Author response: To remove the emphasis from the above term used and provide clarity, we rephrase the sentence as follows:

Lines 211-213: “**We used a moderate emissions scenario only (Representative Concentration Pathway 4.5) given overlapping climate projections across scenarios over the next few decades (43).”**

*Line 220: Why was “a single temperature threshold was used for the entire state rather than thresholds derived from local historical data?” What would the analysis look like if it also used local data? This could get at the regional variation issue raised above.*

Author response: We use three ways to measure extreme heat in this study: daytime heat waves, nighttime heat waves, and heat index days. A daytime or nighttime heat wave is one such that its projected temperature is greater than the census tract’s historical 95^th^ percentile maximum or minimum temperature for two days, respectively. These designations of extreme heat consider a census tract’s local climatology. The heat index does not consider local climatology. As a best practice we included relative (heat waves) and absolute (heat index days) measurements of extreme heat as they present different adaptation issues. Although relative extreme heat is more relevant to determining heat-health impacts, there is value in knowing places that will be hot in an absolute sense in the future. There are physiological and adaptation limits that come with very high temperature extremes.

The heat waves included in this study are locally relevant and enable analysis of how measurements of risk evolve with different understandings of vulnerability and heat exposure.

*Line 277: Why are “vulnerability in communities with low population densities is ranked the highest by SVI and lowest by HHAI?” This would be a great opportunity dig into the reasons behind the differences as opposed to primarily reporting on them as the article does now.*

Author response: We address why the indices rank communities with diverging population densities to some degree in one of the reviewer’s previous comments. The relevant section is reproduced again for reference. Further, we now include a new supplementary figure that sheds additional light on the reasons for differences across the indices, as included below.

Lines 332-342: “The indices differentially prioritize urban versus rural vulnerabilities. Vulnerability in communities with low population densities is ranked the highest by SVI and the lowest by HHAI **(S2 Fig).** However, across indices, tracts with high population densities generally are more vulnerable. Among tracts in the top decile of population density, median vulnerability index percentile values are 75.0 (CES), 86.8 (HHAI) and 78.0 (SVI), while tracts in the bottom decile of population density have median vulnerability index percentile values of 35.7 (CES), 19.5 (HHAI), and 44.0 (SVI). **HHAI includes three indicators that are important to characterizing urban-specific heat vulnerabilities but are less relevant for rural communities: impervious surface coverage, tree canopy coverage, and the change in urbanized land (Table 1). SVI excludes environmental, pollution, and health related indicators, which may make it better able to characterize the drivers of vulnerability in low population density tracts (Table 1 and S2 Fig).**”

Lines 829-833: “**S2 Fig: Vulnerability percentiles are greater in tracts with larger population densities. (a) Average vulnerability percentile (CES, HHAI, SVI) is plotted by log average population density of tracts (people per square kilometer, bin width of 2.5 percentiles, log scale). (b) This plot shows the frequency of different population densities across California’s census tracts (people per square kilometer, log scaled x-axis).”**

*Line 314. Can the authors explain why only “13.7% (1085) of tracts in the state are vulnerable (75th percentile or above) under all three indices?” This seems to be one of most important points of the paper can use more explanation.*

Author response: As mentioned above, it is difficult to separate out the impacts of individual indicators on the overall vulnerability scores for HHAI and SVI because they used principle component analysis. We address the reviewer’s comment as to why there is only minor agreement across the studied indices with the following text below, providing some explanation:

Lines 385-392: “Some communities are considered vulnerable regardless of the index used. 13.4% (1060) of tracts in the state are vulnerable (75th percentile or above) under all three indices **despite differences in indicator selection, measurement of those indicators, and calculation methods (Fig 2). In addition, disagreement across tracts designated as “disadvantaged” likely results from regional differences in vulnerability across the state as well as differences in the overall focus of the indices. “Disadvantaged” designations for tracts vulnerable under all indices point to especially high priority communities, bolstering findings from previous work (33).”**

*Line 449: The authors call for “a holistic approach for adaptation investments.” I agree this is an important point—but what would this look like? How can they use their analysis to make this point more rigorously?*

Author response: We agree with the reviewer that further explanation of a “holistic approach for adaptation investments” is needed. Also, we expand on how the analysis supports the arguments we make. We address this point at the start of the discussion section:

Lines 484-498: “Vulnerability indices are commonly used to prioritize adaptation needs and investments. However, the choice of an index invariably involves trade-offs and challenges. Here, we use extreme heat adaptation in California as a case study to analyze implications for implementing equity-oriented climate adaptations among most vulnerable communities. This study finds that by using CalEnviroScreen 3.0 to designate a census tract’s status as “disadvantaged,” the state does not fully capture community vulnerability to extreme heat, as might be expected. Nuances in the form of extreme heat further obfuscate where adaptation need is greatest given diverging patterns between the type of exposure and vulnerability. We show that, overall, the state has successfully targeted CCI-funded tract interventions to its most “disadvantaged” communities, but that climate change will likely increase the importance of a holistic approach for adaptation investments **that reflects the diversity of the regions, the urban-rural gradient, and the scales of implementation in the state. This work demonstrates that targeted indices (HHAI or CES) and a broader index (SVI) differentially weigh and represent diversity across the large state of California. Therefore on-the-ground validation and integrative applications are vitally important for such quantitative measurements of vulnerability.”**

**Reviewer #3:**

*The paper uses quite a bit of jargon and doesn't define a lot of important terms in terms of their analysis (e.g. vulnerability, index, community). Index is used to refer to both heat indices (which combine meteorological variables) and PCA-based weighting schemes to make an abstract assessment of risk. They include glossary definition in text, but need to better define important terms in a way that is relevant to the paper.*

Author response: We agree that additional clarity is needed around the terms used in this study to prevent confusion. To address the issue, we have gone through and confirm and/or have now added definitions for the following phrases:

- Vulnerability: we included the IPCC definition of vulnerability in the original manuscript. Lines 85-88: “Vulnerability is “the propensity or predisposition to be adversely affected. Vulnerability encompasses a variety of concepts and elements including sensitivity or susceptibility to harm and lack of capacity to cope and adapt” (12).”
- Index: we use the term index to refer to CES, SVI and HHAI. The term “heat index” is the name of an exposure metric developed by the National Weather Service, and we felt that it would be confusing to call it by an alternative name. However, we recognize that it could cause confusion for readers who are less familiar with the heat-health literature and now provide the following clarifying sentence:

Lines 116-117: “**The heat index is not to be confused with a vulnerability index, which is a metric that integrates indicators of vulnerability.”**

- Community: Communities can be defined by many different scales. For the sake of clarity, we now include the following sentence:

Lines 91-93: “**In this study, a “disadvantaged community” is therefore a census tract whose vulnerability index percentile value is in the upper quartile.”**

*In general, the abstract is a bit difficult to read and could use more direct statements about what the paper is going to do. Does CalEnviroScreen 3.0 not consider variables that correlate with heat? What are the two alternative indices? The line "Only 13.7% of communities are vulnerable across all three vulnerability indices studied." seems to indicate that the indices are mutually exclusive?*

Author response: We agree that the abstract would benefit from additional description of the analysis. We have revised it such that it now includes the names of the two alternative indices and emphasizes that CES, the current legally relevant index, is an environmental justice, pollution focused index. To clarify, the statement, “Only 13.7% of communities are vulnerable across all three vulnerability indices studied,” means that that percentage represents the degree to which the three indices overlap. It does not mean that they are mutually exclusive. We include the new abstract with the updates below:

Lines 30-52: “Morbidity and mortality impacts of extreme heat amplified by climate change will be unequally distributed among communities given pre-existing differences in socioeconomic, health, and environmental conditions. Many governments are interested in adaptation policies that target those especially vulnerable to the risks, but there are important questions about how to effectively identify and support communities most in need of heat adaptations. Here, we use an equity-oriented adaptation program from the state of California as a case study to evaluate the implications of **the currently used environmental justice index (CalEnviroScreen 3.0) for the identification of socially vulnerable communities with climate change adaptation needs. As CalEnviroScreen is geared towards air and water pollution, we assess how community heat risks and adaptation needs would be evaluated differently under two more adaptation-relevant vulnerability indices: the Social Vulnerability Index and the Heat-Health Action Index.** Our analysis considers communities at the census tract scale, as well as the patterns emerging at the regional scale. Using the current index, the state designates 25% of its census tracts as “disadvantaged” communities eligible for special adaptation funds. However, an additional 12.6% of the state’s communities could be considered vulnerable if the two other indices were considered instead. Only 13.4% of communities are vulnerable across all three vulnerability indices studied. Choice of vulnerability index shapes statewide trends in extreme heat risk and is linked to a community’s likelihood of receiving heat-related California Climate Investments (CCI) projects. Tracts that are vulnerable under the current pollution-focused index, but not under the heat health specific index, received four times the number of heat-related interventions as tracts vulnerable under the reverse scenario. This study demonstrates important nuances relevant to implementing equity-oriented adaptation and explores the challenges, trade-offs, and opportunities in quantifying vulnerability.”

*line 68: What does "community screening" mean here? Does that mean risk assessment at the community level? Is that sociodemographic or climate-focused? This may benefit from a bit more explanation.*

Author response: The reviewer highlights an important ambiguity in the language we use. We replace “community screening methods” with “vulnerability indices” to clarify the sentence (line 73).

*line 90: May want to also include that heat death is chronically underreported. Gubernot et al 2013 Int J Biometerol & Riley et al 2018 Int J Environ Res Public Health*

Author response: The reviewer highlights a point that would be useful to emphasize further: heat death is chronically underreported and it is likely that health impacts are much larger than they appear in the literature. We address the reviewer’s comment by including the following sentence, citing Gubernot et al. as recommended:

Lines 102-103: **“Reported heat-health morbidity and mortality figures are widely believed to be underestimates, particularly among workers (17,18).”**

*line 138: Can you clarify what "intersected" means for this study? Does this mean that you applied these indices with different heat thresholds? Need more clarity here.*

Author response: We recognize that the term “intersected” is unclear, and we change the sentence of question to refine its meaning:

Lines 157-159: “Second, we **plotted** different definitions of extreme heat **against** these indices to identify statewide relationships between vulnerability and exposure.”

*line 218: 91F is a very low threshold - does this come from one of the cited studies? 91 may be relevant to particularly vulnerable sub-groups (e.g. the elderly and the immunocompromised), but I think you need to better justify why you chose such a low threshold, particularly when much of California is relatively dry.*

Author response: We chose the 91F heat index threshold given prior research that focused on diverse climates of San Diego County. This study found that heat-related health impacts along the coast of San Diego occurred at temperatures as low as 73F. Therefore, the lower bound of the extreme caution range, a heat index value of 91F, was selected to be relevant to coastal and inland areas. To make the justification for the threshold more apparent, we changed the manuscript to the following text:

Lines 240-244: “Previous work has questioned the efficacy of a 105 °F heat index threshold for the state of California (16,25). **Given that health impacts have been shown to emerge at temperatures as low as 73°F for the coastal region of San Diego County, we used the lower bound of the heat index “extreme caution” classification, 91°F, as a mid-range, health-relevant definition for the state (25).”**

*line 279: It seems like tracts with high population densities are less vulnerable on average compared to low density areas? Especially if we don't include Northern California (which is subject to less extreme heat in the first place). The next few lines clarify this a bit, but I think it still could be more clear.*

Author response: In the manuscript we state that “across indices, tracts with high population densities generally are more vulnerable” (lines 3334-335). Therefore, we presume that the reviewer instead means “It seems like tracts with high population densities are **more** vulnerable on average compared to low density areas?” We include an additional supplementary figure (S2 Fig) that further supports this finding (lines 829-833). It is reproduced above for a similar comment made by reviewer 2 so we do not provide it again here. Please see the response above for the text used to introduce it in the manuscript.

*Table 1: This table should be moved earlier in the paper - this would help to clarify the heat-index vs. vulnerability index issue mentioned earlier.*

Author response: We agree with the reviewer that there is an opportunity to improve the narrative flow to improve the readability of the results. Now, the table appears immediately after the first figure and they are discussed together.

*line 345-347: It would be helpful to posit a reason for day/night and inland/coastal differences in heat wave frequency. This plays into your findings for increased heat wave frequency in vulnerable communities as income generally increases towards the coast in California.*

Author response: The reviewer raises a point that is likely of interest to many readers. We include the following information to explanation to elaborate on our findings:

Lines 386-390: “Daytime events will largely impact the inland regions while nighttime exposure will increase along the coasts, in the Inland Desert region, and in the Sierra Nevada region. **Previous work has shown that, for the heat wave probability density functions, geographic variations in tail length and variance lead to such differing daytime and nighttime heatwave predictions (51).”**

*line 449: The use of "holistic" here is vague - this would benefit from more explanation.*

Author response: We agree with the reviewer that the term “holistic” could be further explained to clarify the sentence. Below we include the updated excerpt to which they refer:

Lines 491-498: “We show that, overall, the state has successfully targeted CCI-funded tract interventions to its most “disadvantaged” communities, but that climate change will likely increase the importance of a holistic approach for adaptation investments **that reflects the diversity of the regions, the urban-rural gradient, and the scales of implementation in the state. This work demonstrates that targeted indices (HHAI or CES) and a broader index (SVI) differentially weigh and represent diversity across the large state of California. Therefore on-the-ground validation and integrative applications are vitally important for such quantitative measurements of vulnerability.”**

*line 476: Are all of these indices linear? You've normalized them and then selected "disadvantaged" communities based on a percentile threshold. That may not be valid if they come with specific numeric threshold for identifying disadvantaged communities. You may want to add this to the limitations section.*

Author response: The reviewer raises an important methodological limitation that we agree is useful to list in our discussion section. We include the following sentences in the methods and limitations sections, respectively.

Lines 177-178: “**HHAI scores were converted to percentile ranks for ease of comparison to the other indices.”**

Lines 589-590: “**Further, HHAI is not a percentile-based index so our framing of the “disadvantaged” threshold was for comparison purposes only.”**
